# Supplementary material for: Gradient of tactile properties in the rat whisker pad
Source: PLoS Biol. 2020 Oct 22;18(10):e3000699. doi: 10.1371/journal.pbio.3000699 (PMC7608947; doi:10.1371/journal.pbio.3000699)
Supplement: S1 Fig — (A) To examine to robustness of the map in Fig 1, we changed the distance of the wheel to the pad (texture 5 mm closer to the pad; upper left panel), and wheel velocity (velocity approximately 50 mm/s; upper right panel). The lower panels show the mean and SD of each arc in the upper panels. The inequality sign indicates a statistically significant differences between the various arcs. The underlying data for this Figure can be found in S1 Data. (DOCX) [file pbio.3000699.s001.docx]

Gradient of Tactile Properties in the Rat Whisker Pad

Figures S1

**Erez Gugig^#^, Hariom Sharma^#^, and Rony Azouz**

Department of Physiology and Cell Biology, Zlotowski Center for Neuroscience,

Ben-Gurion University of the Negev, Israel.

# contributed equally

**
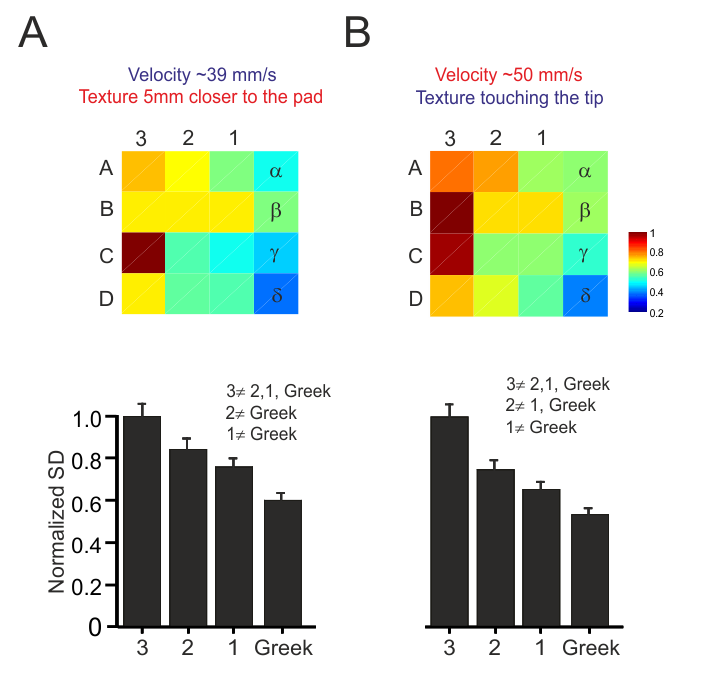
**

Figure S1. Robustness of whiskers' properties map. (A). To examine to robustness of the map in Fig. 1, we changed the distance of the wheel to the pad (Texture 5mm closer to the pad; upper left panel), and wheel velocity (Velocity ~50 mm/s; upper right panel). The lower panels show the mean and SD of each arc in the upper panels. The inequality sign indicates a statistically significant differences between the various arcs. The underlying data for this Figure can be found in S1 Data

To examine the robustness of the caudal-rostral functional gradient that arise from the biomechanical differences of the whiskers, we changed velocity and the proximity of the wheel to the pad. We found that decreasing the distance between moving textures and the pad resulted in a modification of the map, while keeping the rostral-caudal gradient nearly constant (Fig. S1A). Similarly, when increasing texture velocity, we saw minor changes in the map; nevertheless, there were no major changes in the rostral-caudal gradient (Fig. S1B). In both of these conditions, we collapsed all values in each arc across all animals and found statistically significant differences between most arcs (Fig. S1A-B lower panels).
