# Supplementary material for: Gradient of tactile properties in the rat whisker pad
Source: PLoS Biol. 2020 Oct 22;18(10):e3000699. doi: 10.1371/journal.pbio.3000699 (PMC7608947; doi:10.1371/journal.pbio.3000699)
Supplement: S2 Fig — (A) The panel shows the recorded rows. (B) The panels show the mean and SD of each of rows in panel A at different distances from the pad and at different wheel velocity. The biomechanical properties of the whiskers across the different rows and in the different conditions are not significantly different from each other. The underlying data for this Figure can be found in S1 Data. (DOCX) [file pbio.3000699.s002.docx]

Gradient of Tactile Properties in the Rat Whisker Pad

Figure S2

**Erez Gugig^#^, Hariom Sharma^#^, and Rony Azouz**

Department of Physiology and Cell Biology, Zlotowski Center for Neuroscience,

Ben-Gurion University of the Negev, Israel.

# contributed equally

**
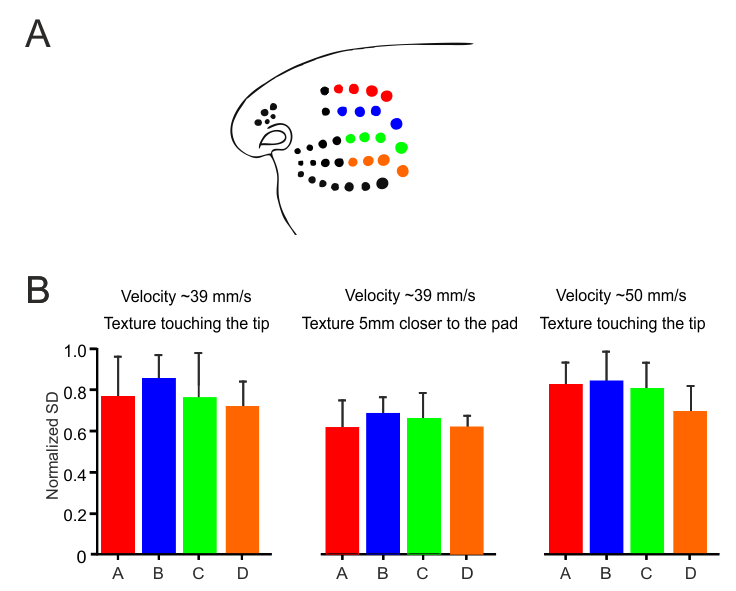
**

Figure S2. Mechanical characteristics of different whiskers rows are not different from each other. (A). The panel shows the recorded rows. (B). The panels show the mean and SD of each of rows in (A) at different distances from the pad and at different wheel velocity. The biomechanical properties of the whiskers across the different rows and in the different conditions are not significantly different from each other. The underlying data for this Figure can be found in S1 Data

To examine whether this gradient exists in the dorsal-ventral plane, we repeated the same analysis by averaging across rows. Fig. S2 shows that the whiskers along the rows are not significantly different in their responses to textures.
