# Supplementary material for: Gradient of tactile properties in the rat whisker pad
Source: PLoS Biol. 2020 Oct 22;18(10):e3000699. doi: 10.1371/journal.pbio.3000699 (PMC7608947; doi:10.1371/journal.pbio.3000699)
Supplement: S3 Fig — (DOCX) [file pbio.3000699.s003.docx]

Gradient of Tactile Properties in the Rat Whisker Pad

Figures S3

**Erez Gugig^#^, Hariom Sharma^#^, and Rony Azouz**

Department of Physiology and Cell Biology, Zlotowski Center for Neuroscience,

Ben-Gurion University of the Negev, Israel.

# contributed equally

**
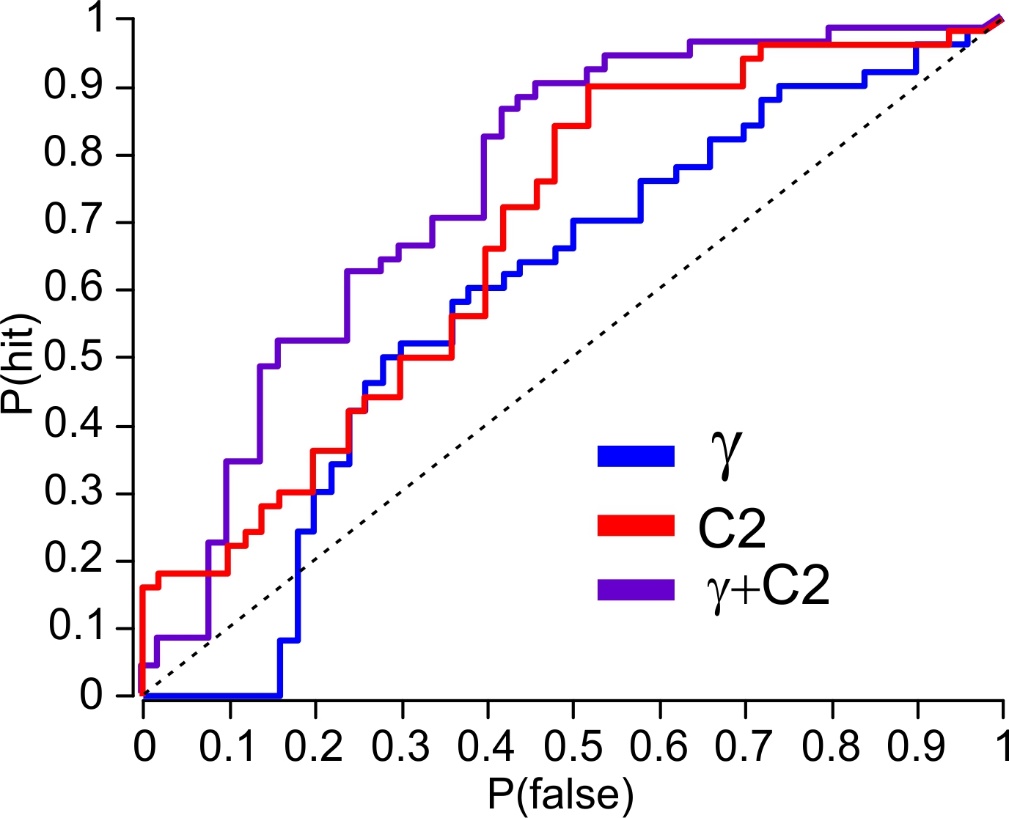
**

Figure S3. ROC curve for discrimination between P120 and P220 textures for γ (blue), C2 (red) and their combination.
