# Supplementary material for: Gradient of tactile properties in the rat whisker pad
Source: PLoS Biol. 2020 Oct 22;18(10):e3000699. doi: 10.1371/journal.pbio.3000699 (PMC7608947; doi:10.1371/journal.pbio.3000699)
Supplement: S4 Fig — (A) Similar to Fig 7A, but textures are 5 mm closer to the pad. (B) AUC for texture discrimination (blue bars) and texture-edge discrimination (red bars) for the different arcs. (C) Similar to panel A but for rows. (D) Similar to panel B but for rows. The numbers indicate the different arcs. The inequality sign indicates a statistically significant difference between the various arcs. The underlying data for this Figure can be found in S1 Data. (DOCX) [file pbio.3000699.s004.docx]

Gradient of Tactile Properties in the Rat Whisker Pad

Figure S4

**Erez Gugig^#^, Hariom Sharma^#^, and Rony Azouz**

Department of Physiology and Cell Biology, Zlotowski Center for Neuroscience,

Ben-Gurion University of the Negev, Israel.

# contributed equally

**
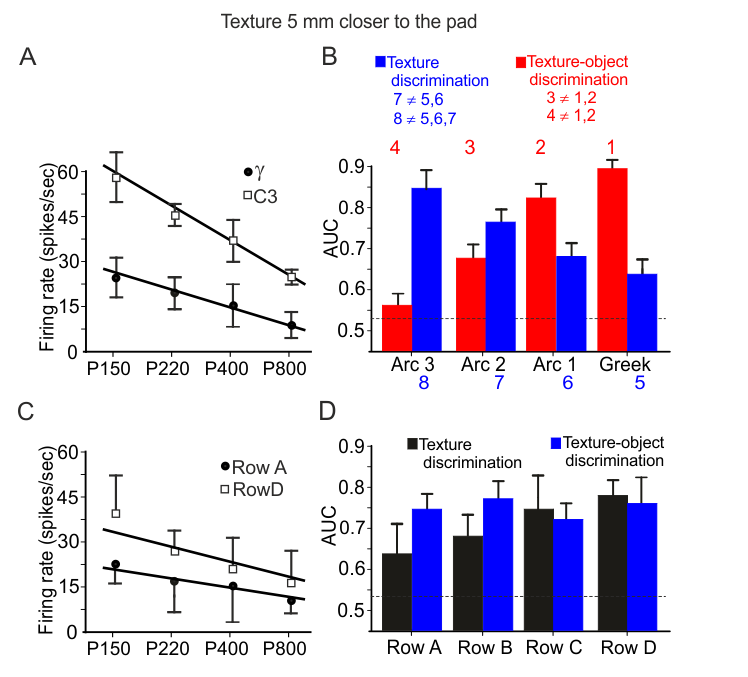
**

Figure S4. A robust gradient of textures and edge-texture discrimination capabilities in TG neurons. (A). Similar to Fig. 7A but textures are 5 mm closer to the pad. (B). AUC for texture discrimination (blue bars) and texture-edge discrimination (red bars) for the different arcs. (C). Similar to A but for rows. (D). Similar to B but for rows. The numbers indicate the different arcs. The inequality sign indicates a statistically significant differences between the various arcs. The underlying data for this Figure can be found in S1 Data

To examine the robustness of our findings that neurons innervating rostral whiskers are better suited for discriminating between textures, we repeated the experiments in Fig. 7 in partially different set of whiskers at a closer distance of the wheel to the pad (Fig. S4D). While we observed an increase in the firing rates (Fig. S3), the gradient in texture and edge-texture discrimination persisted.

To examine the role of the different *rows* in texture and texture-edge discrimination. Fig. S4C-D shows that A3 neuron has a steeper dependence of firing rates on texture coarseness than D3 neuron. However, averaging across all neurons revealed that neurons in the different rows do not differ in their texture discrimination and edge-texture discrimination (Fig. S4D).
