# Supplementary material for: Gradient of tactile properties in the rat whisker pad
Source: PLoS Biol. 2020 Oct 22;18(10):e3000699. doi: 10.1371/journal.pbio.3000699 (PMC7608947; doi:10.1371/journal.pbio.3000699)
Supplement: Attachment — Submitted filename: respone_to reviewers1_ver2.docx [file pbio.3000699.s006.docx]

June 15^th^, 2020

Senior Editor

PLOS Biology

Dear Dr. Roberts:

Thank you for your letter dated Apr. 18^th^ and the attached review of our manuscript (PBIOLOGY-D-20-00397R1) entitled "Gradient of Tactile Properties in the Rat Whisker Pad".

We have carefully addressed each of the concerns raised by the reviewers, modified the manuscript extensively, and would now like for you to reconsider the paper for publication in *Plos Biology*. The reviewer’s comments and our responses to them are detailed below, and the revised manuscript (marked in red in the text) and figures are also enclosed.

We look forward to your decision on the paper. Thank you very much for your time and effort.

Sincerely,

Rony Azouz, Ph.D.

We thank the reviewers for their extensive and careful evaluation of the paper. The clarity and accuracy of the paper has been greatly improved as a result. Our response to each of the issues raised is given below.

**Response to reviewer #1.**

MAJOR

***Comment 1:*** There is minimal presentation of raw data: It would be useful to include raster plots of neuronal spiking activity as the whiskers interact with the edges and the textures. (These could be aligned to the contact with the edge, similar to the PSTHs in Figure 5A.) This could be included for a few example neurons, and would provide the reader with an idea of trial to trial variability in the neuronal response to a particular texture. In Figure 1, it would be nice to include an inset to show the vibrations induced by the two textures at much higher temporal resolution.

***Response:*** We agree with reviewer and now added more raw data traces in all relevant figures.

***Comment 2:*** A clearer justification for using the normalized SD of vibrations. I am confused by the fact that the highest values of normalized SD in Figure 4C are higher than 1. This is particularly apparent for the green and red curves.

***Response:*** We thank the reviewer for noticing this fact. We now corrected it and elaborate on it in the text. We chose to normalized the SD signals since we were recording from different animals at different ages with large changes in whiskers signal. We always normalized the values to the maximal value in each animal (in some animals we used the two sides of the face, hence the error bar).

***Comment 3:*** Similarly, for Figure 4D the use of "normalized" slope needs better justification.

***Response:*** See response to previous comment. Since we care about the relative and not absolute signals, we normalized the slopes to the maximal slope. The largest slope value was in all cases ARC4 and we normalized all arcs in relation to this. We now elaborate in the manuscript.

***Comment 4:*** The statistical analysis related to Figure 6D presented in the form of inequality signs are not clear and difficult to understand.

**Response:** We have modified the figure and legend to make it clearer.

***Comment 5:*** I found the video analysis in Figure 8, the least convincing part of the manuscript. The authors argue that when the rat approaches a small food pellet, the rostral whiskers are directed forward, whereas caudal whiskers point more backward. Presumably the rat is approaching the small food pellet to sniff it or to put it in its mouth. In which case it makes sense for the most rostral whiskers to be directed forward to sense the pellet as the rat moves towards the pellet. It is difficult to conclude from this observation that the rat would use its rostral whiskers to examine any texture. For example, the situation might have been different if the rats were to examine the textured walls while running through a tunnel.

***Response*:** We thank the reviewer for his comment and have decided that this analysis and figure is not necessary for the message we want to covey. We took out the figure and related analysis from the paper.

**Minor:**

***Comment 6:*** There are a number of typos that should be corrected by a more careful proof reading. For example:

Page 10; Second paragraph:

The panel show that the range of ...

... the rostral whisker respond with a higher range of ...

This implies for all whiskers.

Page 12; Second paragraph

We found a steeper slope for the rostral whisker then the caudal whisker,

Legend for Figure 1C

An heat map ...

Legend for Figure 6D

The inequality sign indicates a statistically significant differences between the various arcs.

***Response:*** We corrected all typos mentioned and scan through the paper with a fine toothcomb.

***Comment 7:*** The use of "object" and "edge" are at times confusing. For example, in Figure 1A you indicate "Edge", and in 1B, this is referred to as "Object". I think a consistent usage of "edge" would be clearer.

***Response:*** We modified the paper so only the phrase "edge" appears.

**Response to reviewer #2.**

MAJOR

***Comment 1:*** While I did appreciate the contents of the paper, the authors are overlooking one substantial form of analysis that would potentially give much more meaning to their results. The issue is introduced by this sentence: "One major feature that stem from our results is that within a single whisk, rats may sample a region of an object at multiple spatiotemporal scales simultaneously." As far as I could tell (and correct me if wrong, and then ignore this point) the investigators did no analysis of combined signals from multiple whiskers. I most strongly recommend doing ROC or their preferred decoding method where the observer attempts to identify the texture using the signal from 1 whisker alone, where the whisker is positioned from rostral to caudal. Now with 2 whiskers, where both whiskers have the same location (the same exact whisker twice, as a control comparison) and where the 2 whiskers have different positions. Same for 3, 4, 5 whiskers. Analyze how the decoding performance increases with number of whiskers, and test whether whisker DIVERSITY makes a difference. For instance, a rostral whisker alone may carry a better signal than a caudal whisker, but caudal plus rostral may carry a supralinearly high signal. And maybe 1 caudal whisker signal combines with 1 rostral whisker signal better than 1 rostral plus 1 rostral. In other words, diversity enriches the potential combinations in highly dimensional space and caudal whiskers might do more than supposed when looked at singly.

***Response:*** We thank the reviewer for this comment and added a new section and corresponding figure (Fig. 4D-F) examining this issue. Indeed, we found distance-dependent supralinear summation of whisker signals.

INTRO

"Facial whiskers, or vibrissae, are found in many mammalian species. They project outwards and forwards from the nose of the animal to form a tactile sensory array that surrounds the head [1].

***Comment 1:*** have heard that it is not just MANY but ALL mammals except humans, but I do not have a scientific source for that.

***Response:*** Me too, but unfortunately, I can only report what I know from the literature.

***Comment 2:*** Nose: I do not think this is correct … cheeks, chin, … "snout" is a common term… not nose. "non-moveable" non-mobile would be a more correct term."

***Response:*** Corrected*.*

***Comment 3:*** Rodents use their whiskers to detect and distinguish a variety of tactile features in their environment [8] including edge position [9, 10], shape [5, 11], aperture and gap width [12], and texture discrimination [7, 13-18]."

Eliminate final word, "discrimination" - redundant with "detect and distinguish"

***Response:*** Corrected.

***Comment 3:*** An extension of this concept lies also in the "kinetic-signature" hypothesis [21, 27, 28] …"As far as I remember, the term "kinetic signature" was not ever used in relation to a whisker's intrinsic properties, but in relation to the distinct motion caused by contact with different surfaces. I thought the signature was of the stimulus, not the whisker. I think the hypothesis of "splitting the tactile vibration signals into labeled frequency lines in the cortex" is quite different from the "kinetic signature" hypothesis. I may be wrong but I would like the authors to re-check.

***Response:*** We thank the reviewer for pointing our mistake. We have rewritten this entire introduction section.

METHODS

***Comment 3:*** *"*Each revolution of the wheel lasted for about 3 sec. and no noticeable adaptation in neuronal firing rates were detected." Please comment on whether this disagrees with Hartmann claim of some adapting neurons in the ganglion. In fact, later (Results) we have: "These firing patterns are conventionally used to characterize neurons as either rapidly adapting (RA) or slowly adapting (SA), respectively. We did not find any significant bias in neuronal types towards any of the arcs (Table 1)."

***Response:*** It should be noted that that we are using different types of stimuli. The first is just whisker movement. In this type of stimuli, we do see adaptation since we see RA and SA neurons. We claim that there are no spatial relations between cell types and location on the pad. Whereas the second stimuli involve rotating wheel covered with textures pressing against the whiskers causing variable lateral force and bending moment applied for each whisker

***Comment 4:*** *"*To examine the influence of whisker identity on responses to edges and textures (Fig. 1A,B), we separated edge and texture related stimulation epochs by identifying big excursion (mean±3SD) in whisker vibrations during wheel rotation." Is this a circular argument? I mean, are the authors deciding a priori what form of excursion whiskers must use in order to encode textures and edges?

***Response:*** In some of the experiments, we recorded with a high-frame rate video camera the contact of whiskers with texture and edges. A larger excursion of whisker movement was always associated with edges. 3SD was a criterion that could distinguish in all cases edges from surfaces. We now add this into the METHODS section.

RESULTS

***Comment 5:*** *"*Vibrissae movements were measured in response to sandpapers having two different grades: P220, P400. However, since we got similar results for all textures (not shown), we combined all these results together."This does not make sense at all, to me. Combined what? Please clarify the sentence.

***Response:*** We apologize for the confusion caused by our description. We created a map for P220 and for P400. Since we got similar maps for these textures (not shown), each pixel in the map is a reflection of an average of multiple whiskers responding to these two textures. We modified the text accordingly.

***Comment 6:*** Please give more informative labels to Figure 1C-D.

***Response:*** Done*.*

DISCUSSION

***Comment 7:*** In this scheme, longer caudal whiskers transmit mainly "where" information, whereas rostral shorter vibrissae transmit both "where" and "what" information."

This is a commonly made statement, going back to Brecht et al. 1997 and it may be valid when rats have full access to the object of interest with all whiskers. But rats are frequently "reachers," e.g. extending themselves from one step to feel the one below on the stairway down to the subway station. When they cannot contact with micros, they may very well use macros for texture and other fine judgments. In fact many behavioral experiments show excellent texture discriminations with macros, when the rat can reach only with those.

***Response***: We agree with the statement of the reviewer. We don't say as Brecht that there is a dichotomy between micro and macro, rather there is a gradient in macros in which the most caudal whiskers transmit ***mainly*** "what" information whereas the more rostral whiskers transmit both. We have rephrased the paragraph to make it clearer

***Comment 8:*** The paragraph beginning

"We found that whiskers, which are the first stage of tactile information translation, form a location- dependent gradient in which rostral whiskers show smaller attenuation in the transformation from texture coarseness to whisker vibrations…"

is a mini-review of texture coding by the whiskers, and in that context the authors will be interested in a recent paper, Zuo et al. CurrBiol "Rats Generate Vibrissal Sensory Evidence until Boundary Crossing Triggers a Decision" which breaks down some of the whisker kinematic features that distinguish texture.

***Response:*** Thanks. We now include this a in all new section in the discussion.

***Comment 8:*** There are several misspellings and typos throughout the paper, e.g. "criterions". I have not listed them.

***Response:*** We corrected all typos mentioned and scan through the paper with a fine toothcomb

**Response to reviewer #3.**

***Comment 1:*** I believe this paper would have greater impact if the less groundbreaking (1-3) or less developed (8) parts of it were condensed into fewer figures or eliminated. A more compact presentation of only the key data pieces needed to support the main punchline of discrimination gradient, plus the existing detailed characterization of the TG gradient would bring the strengths of the work to the forefront.

***Response:*** We thank the reviewer for his comments. We moved some of the panels in Fig. 1-3 to supporting figures and kept just the main ideas. Further we extended the analysis in Figure 4 into curvature and the properties of the power spectra of whisker position.

***Comment 2:*** Figs 1 & 2. The shorter whiskers had higher positional variance (SD) and deflection from the edge during the rotation of the texture wheel. Bigger whiskers have more inertia, so the same level of force might be expected to move them less. Yet bigger whiskers also sweep across a wider range of motion, which could exert more force on objects during active whisking. Would increased force applied by the bigger whiskers during whisking be sufficient to normalized the level of vibration across the grid?

***Response:*** We thank the reviewer for this comment. We've tested for this notion in new Fig 5 by making surface distance to the pad constant, so the force on the caudal whiskers is bigger. We also measure whisker curvature which is a better indication of the forces exerted on the follicle. We found that irrespective of the distance, still the variance in the rostral whiskers is bigger. We now describe it the text related to Fig. 5

***Comment 3******:*** Figure 2 varies applied force by changing distance of wheel from the face and shows the same gradient of sensitivity exists, but this doesn't quite address the question, as the force change is uncontrolled for each whisker. How was the force applied by the wheel calibrated across the grid? These results would be strengthened by a more rigorous calculation of lateral force and bending moment applied for each whisker position. The overall lateral force and bending moment could likely be computed from known whisker mechanical properties (see work by Hartmann, Svoboda, Golomb) and the degree of curvature change from videos that have already been collected.

***Response:*** See response to previous comment*.* We have repeated the analysis with whisker curvature and found that it does affect our conclusions. We modified the figure and text accordingly.

***Comment 4:*** Figure 3 makes a fairly sweeping claim that "Mechanical characteristics of different whiskers rows are not different from each other." Even with the text results limiting this to "responses to textures" it seems that this claim is more a reflection of the coarse measurement approach (SD) rather than the whiskers themselves. Certainly, whiskers that have varying lengths or diameters will have different vibrational characteristics that are not captured in this one-dimensional characterization (e.g. classic work by Chris Moore, or more recent computational studies by Shulz, Debrégeas and Wandersman, or Hires and Efros). However, SD may be a sufficient level of characterization for firing rate base texture discrimination models used in the TG recording analysis.

***Response:*** We agree the given the simplicity of our analysis of using the SD, we might be missing some other aspects of tactile transmission. However, we believe we dealt with the finding by Chris Moore and we're not sure how relevant the work by Hires and Efros to texture. We agree that the study of Yves Boubenec et. al. is groundbreaking. We now added a section in the discussion in the paragraph dealing with the Methodological considerations in the study and make reservation about our conclusions.

***Comment 5:*** Fig 4. I'm not sure that using different scale bars in A is worth it. While blowing up gamma lets you see the variance a bit better, the other key point authors are making is in the differences in vibration amplitude. It would be more visually compelling with gamma having the same scale bar as C4.

***Response:*** Done.

***Comment 6:*** Furthermore, the single trial data does not appear to match 4B. After normalizing scale bars, gamma looks about half the SD as C4 in A, but looks <0.1 the SD of C4 in B. What is the reason for that discrepancy? Also, why is B y-axis starting at 0.05 instead of 0?

***Response:*** We thank the reviewer for noticing this discrepancy between the panels. The fault was with the scale bar in the left panels. Moreover, the panels on the left reflect a short segment in time whereas the panel on the right reflects an average of many segment. We corrected the two panels.

***Comment 7:*** Fig 5 This is the key figure in the paper and it is a quite striking result. The longer whiskers clearly are insensitive to texture but retain robust edge responses. On the other hand, the edge responses are hard to pick out from texture responses in the short whiskers. 5A is a beautiful visualization of this gradient. Now the benefit of the experimental apparatus with both texture and edge stimuli is clear.

***Response:*** We thank the reviewer for this comment.

***Comment 8:*** Fig 6&7 Are a nice extension of the results in 5 showing the discrimination gradients are arc not row dependent. Fig 7 is somewhat repetitive with Fig 6. Is there a way to demonstrate the robustness with fewer panels?

***Response:*** The figure shows that dependence on are robust irrespective of distance as well as row dependence. We moved this figure to supplemental material.

***Comment 9:*** Fig 8. This figure adds little and should either be significantly improved or eliminated. The repetition of all 6 mice that basically shows the same thing serves little purpose. It's obvious that caudal whiskers point more backward then rostal ones, that's how the follicle array is designed. Is there something special that happens when the animal is foraging that isn't apparent when looking at a rat at rest? It's hard to understand how the analysis is being performed. It's confusing to use SD as a metric for whisking angle when it was previously used for vibration range. Why not use field standard approaches to measuring amplitude with a Hilbert transform? At least label the y-axis something like "Whisking amplitude (SD)" Some annotated single trial whisker angle time series data between A and the rest of the figure would help guide the reader.

***Response:*** We thank the reviewer for his comment and have decided that this analysis and figure is not necessary for the message we want to covey. We took out the figure and related analysis from the paper.
