# Supplementary material for: Gradient of tactile properties in the rat whisker pad
Source: PLoS Biol. 2020 Oct 22;18(10):e3000699. doi: 10.1371/journal.pbio.3000699 (PMC7608947; doi:10.1371/journal.pbio.3000699)
Supplement: Attachment — Submitted filename: respone_to reviewers3.docx [file pbio.3000699.s007.docx]

Aug. 16^th^, 2020

Senior Editor

PLOS Biology

Dear Dr. Roberts:

Thank you for your letter dated Aug. 3^rd^ and the attached review of our manuscript (PBIOLOGY-D-20-00397R2) entitled "Gradient of Tactile Properties in the Rat Whisker Pad". We have carefully addressed each of the concerns raised by the third reviewer, modified the manuscript, and would now like for you to reconsider the paper for publication in *Plos Biology*. The reviewer’s comments and our responses to them are detailed below, and the revised manuscript (marked in red in the text) and figures are also enclosed.

We look forward to your decision on the paper. Thank you very much for your time and effort.

Sincerely,

Rony Azouz, Ph.D.

**Response to reviewer #3.**

***Comment 1:*** The new figures on supralinear summation are interesting. The AUC data in Figure 4 would benefit from the display of an example ROC curve for one whisker. The multiwhisker analysis would benefit from display of individual ROC curves for two whiskers overlaid with the ROC curve for their combined discrimination power.

***Response:*** Since the relevant figure 4 is already cramped, we added an ROC curve in a new supplementary figure 3.

***Comment 2:*** Some figures are still hard to understand at a glance. This could be alleviated with judicious labelling within the figures. For example, 1B could be labelled "Whisker vibrations", 1C could be be labelled "Texture response." in 1D the label "Normalized SD" is more a designation of units, not of the feature being measured (Whisker vibrations). The authors could improve the readability of their manuscript by examining each figure subpanel and giving them the best, succinct label. Figure 2 is better in this regard, but many other figures plots are not optimally labelled.

***Response:*** We thank the reviewer. We now corrected it and added labels to most subplots.

***Comment 3:*** Likewise, all of the figure titles could be improved. I recommend making the figure title be an accurate take home message of the figure, rather than a restating of the plots. For example, instead of "Response sensitivity of the different whisker arcs to different textures", a better title would be "Shorter whiskers exhibit higher response sensitivity to texture grain". Priming the reader with titles in that style for all figures will help them more quickly grasp the result.

***Response:*** We have modified most figures captions.

***Comment 4:*** *Intro* I recommend the following tweaks " thereby differentially influencing "

" of each individual whiskers "

**Response:** corrected.
